# 4,4’-(Ethene-1,2-diyl)dipyridinium bis(2-hy­droxy-3-meth­oxy­benzoate)

**DOI:** 10.1107/S2414314622005107

**Published:** 2022-05-17

**Authors:** Devin J. Angevine, Jason B. Benedict

**Affiliations:** a Department of Chemistry, The State University of New York at Buffalo, Buffalo, New York 14260-3000, USA; University of Aberdeen, Scotland

**Keywords:** crystal structure, organic co-crystal, vanillic acid, bi­pyridine ethyl­ene

## Abstract

In the title double proton-transfer salt, C_12_H_12_N_2_^2+^·2C_8_H_7_O_4_^−^, consisting of a 1:2 ratio of 4,4’-(ethene-1,2-diyl)dipyridinium cations (*trans* bipyridinium ethyl­ene) to 2-hy­droxy-3-meth­oxy­benzoate anions (*o*-vanillate), the complete cation is generated by crystallographic inversion symmetry and it is linked to adjacent *o*-vanillate anions by N—H⋯O hydrogen bonds, forming trimolecular assemblies. The trimers are linked by C—H⋯O hydrogen bonds as well as aromatic π–π stacking inter­actions into a three-dimensional network. The anion features an intra­molecular O—H⋯O hydrogen bond.

## Abstract 2

In the title mol­ecular salt, the components are linked by N—H⋯O and C—H⋯O hydrogen bonds.

## Graphical abstract

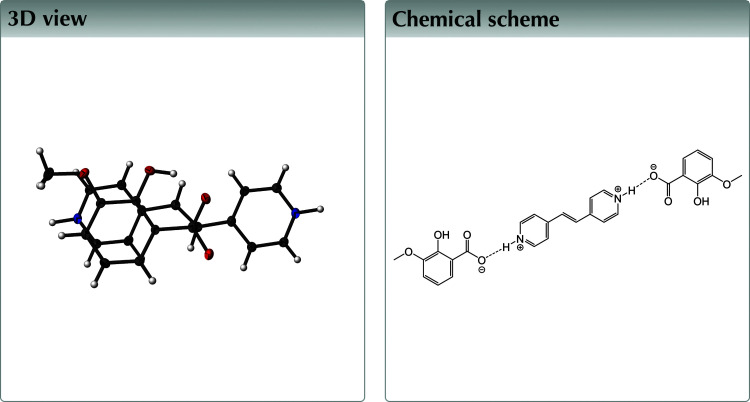

## Structure description

2-Hy­droxy-3-meth­oxy­benzoic acid (*o*-vanillic acid, C_7_H_8_O_4_) is similar in nature to its isomeric counterpart 4-hy­droxy-3-meth­oxy­benzoic acid (*p*-vanillic acid), with the exception of the hydroxyl-group positioning. Much like its counterpart, *o*-vanillic acid is being investigated for its medicinal benefits, such as its anti-allergic inflammatory effects (Kim *et al.*, 2017 ▸). Despite its potential usage for medicinal purposes, there is a significant lack of structural data on this compound and its salts. As such it is beneficial to study the solid-state forms of *o*-vanillic acid and its salts to better understand its inter­actions. To achieve this, bi­pyridine ethyl­ene (C_12_H_10_N_2_) was selected due to its demonstrated ability to form both simple and complex hydrogen-bonded networks (MacGillivray *et al.*, 2000 ▸; Wang *et al.*, 2007 ▸). In addition, as the Δp*K*_a_ value between *o*-vanillic acid (p*K*_a_ = 2.5) and bi­pyridine ethyl­ene (p*K*_a_ = 5.5) is approximately 3, the observed salt formation can reasonably be expected due to the acid–base crystalline complexes Δp*K*_a_ rule (Cruz-Cabeza, 2012 ▸).

**Figure 1:**
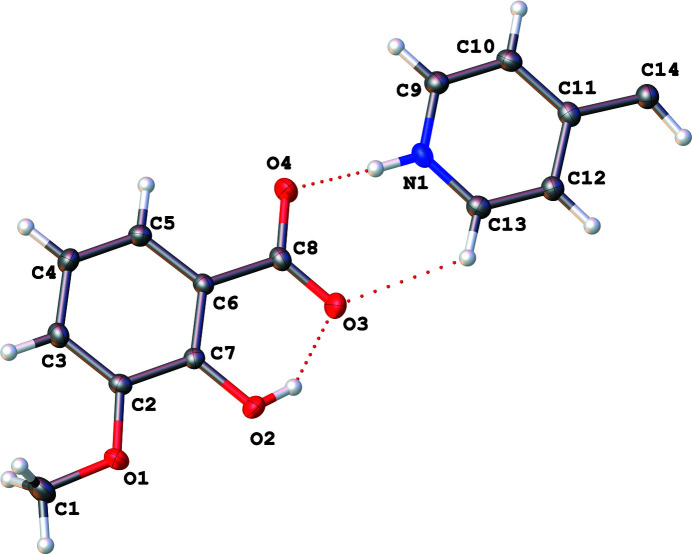
The asymmetric unit of the title mol­ecular salt showing 50% displacement ellipsoids.

**Figure 2:**
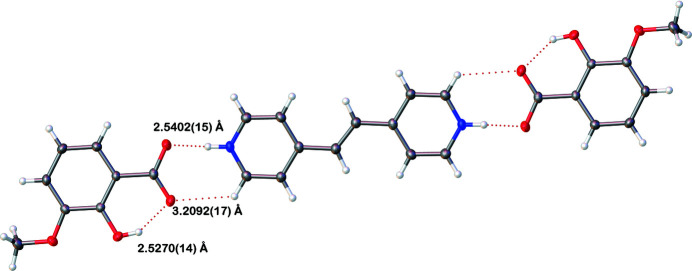
A trimolecular unit of the synthesized salt consisting of two *o*-vanillate anions and one bipyridinium ethyl­ene cation. Hydrogen-bonding inter­actions are shown as red dashed lines with distances displayed between inter­acting heteroatoms.

**Figure 3:**
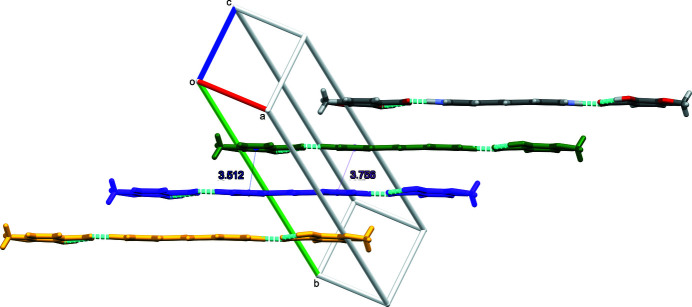
Side on view of the π-stacked trimolecular units. Offset units are shown in different colors. Distances, in Å, are shown between inter­acting ring centroids.

**Figure 5:**
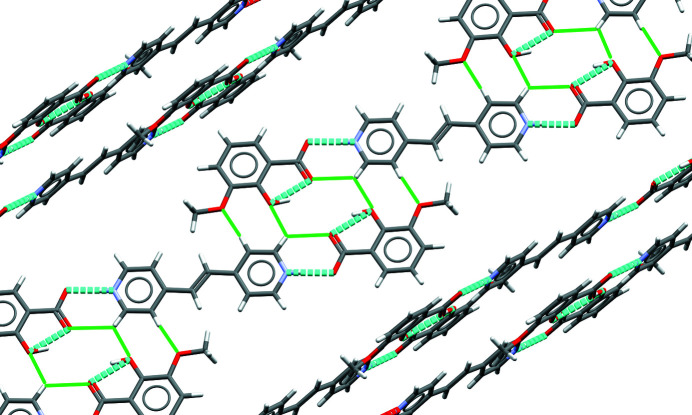
A single layer of the side by side trimolecular unit inter­actions are shown. Hydrogen-bonding inter­actions are shown as dashed blue lines; C—H⋯O type hydrogen-bonding inter­actions are shown as solid green lines.

**Figure 6:**
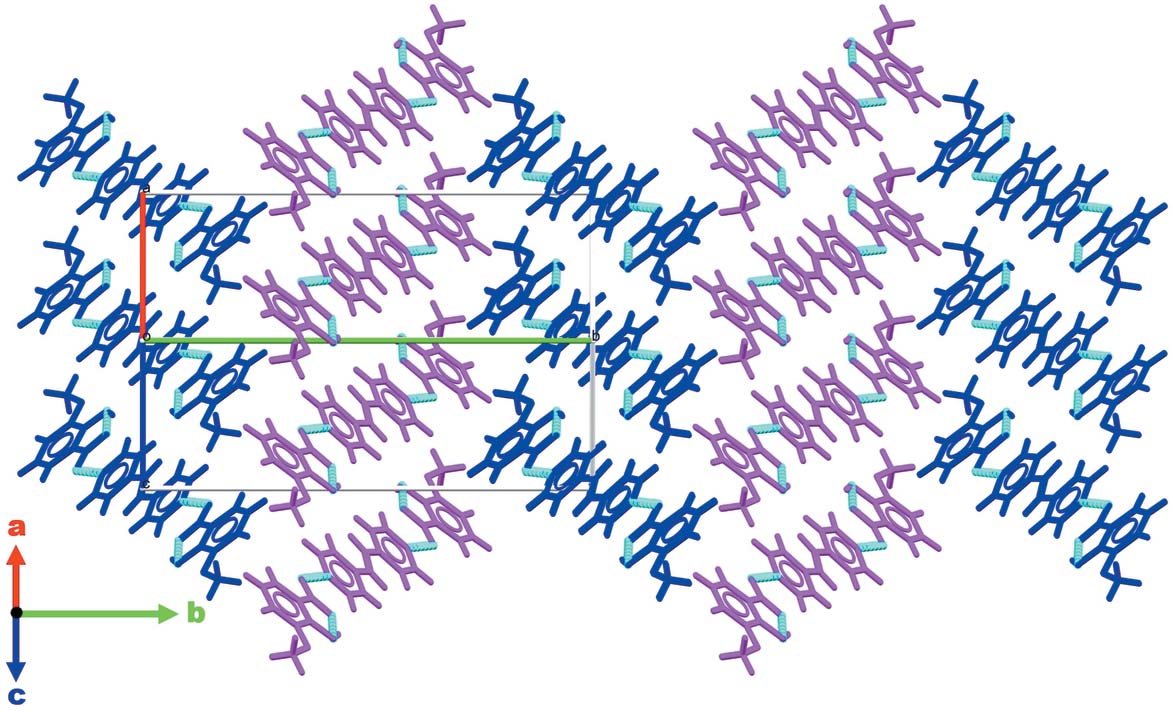
View down [101] showing slipped stacks running along [101] with alternating domains parallel to [010] being highlighted in pink and blue. Hydrogen-bonding inter­actions are shown as blue dashed lines.

The structure of the resulting bipyridinium ethyl­ene bis-*o*-vanillate mol­ecular salt, C_12_H_12_N_2_^2+^·2C_8_H_7_O_4_^−^, exhibits monoclinic (*P*2_1_/*c*) symmetry at 90 K: the complete cation is generated by crystallographic inversion symmetry. A trimolecular unit consisting of one bipyridinium ethyl­ene cation (BPyE) with two *o*-vanillate anions, each of which accepts an N1—H1⋯O4 hydrogen bond from the pyridinium N atoms of the cation is observed, in which the H1⋯O4 distance of 1.45 (2) Å and the N1⋯O4 separation of 2.5402 (15) Å are notably short. The cation–anion bonding is consolidated by a C13—H13⋯O3 link and within the anion, an *S*(6) intra­molecular O2—H2⋯O3 hydrogen bond is observed between the hydroxyl group and the O atom of the carboxyl group (Fig. 1 ▸, Table 1 ▸). These trimolecular units (Fig. 2 ▸) then stack through aromatic π–π inter­actions [shortest centroid–centroid separation = 3.5125 (11) Å between the N1/C9–C13 and C2–C7 rings] with an approximately one third unit offset (Figs. 3 ▸ and 4 ▸). The stacks then sit aside of an alternating stack of units and are cross-linked through C—H⋯O type hydrogen bonds (Fig. 5 ▸). When viewed down [101], the slipped stacks can be seen running along [101], with alternating domains parallel to [010] (Fig. 6 ▸).

## Synthesis and crystallization

A 1:2 molar ratio of bi­pyridine ethyl­ene (182.2 mg, 1 mmol) and *o*-vanillic acid (336.2 mg, 2 mmol) were dissolved into a vial of excess methanol. The resulting solution was vortexed for 30 s at 3,000 rpm on a VWR Mini Vortexer MV I. The solution was then stored in the dark uncapped to allow for crystal formation while the solvent slowly evaporated.

## Refinement

Crystal data, data collection, and structure refinement details are summarized in Table 2 ▸.

## Supplementary Material

Crystal structure: contains datablock(s) I. DOI: 10.1107/S2414314622005107/hb4406sup1.cif

Structure factors: contains datablock(s) I. DOI: 10.1107/S2414314622005107/hb4406Isup2.hkl

CCDC reference: 2172156

Additional supporting information:  crystallographic information; 3D view; checkCIF report

## Figures and Tables

**Figure 4:**
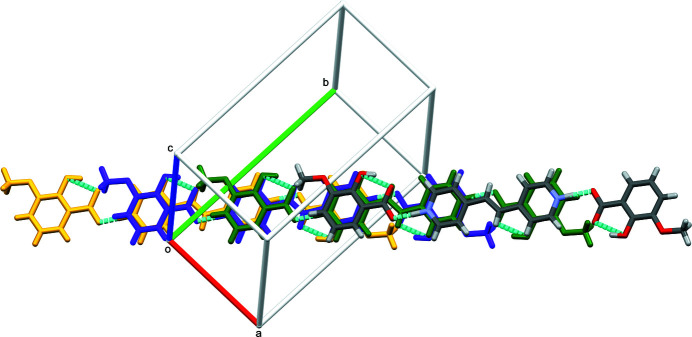
Top down view of the π-stacked trimolecular units. Offset units are shown in different colors.

**Table 1:** Hydrogen-bond geometry (Å, °)

*D*—H⋯*A*	*D*—H	H⋯*A*	*D*⋯*A*	*D*—H⋯*A*
N1—H1⋯O4^i^	1.09 (2)	1.45 (2)	2.5402 (15)	178 (2)
O2—H2⋯O3	0.96 (2)	1.64 (2)	2.5270 (14)	150 (2)
C13—H13⋯O2^ii^	0.95	2.52	3.1831 (16)	127
C13—H13⋯O3^i^	0.95	2.57	3.2092 (17)	125
C12—H12⋯O1^ii^	0.95	2.46	3.3591 (17)	159

Symmetry codes: (i) 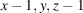 ; (ii) 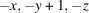 .

**Table 2:** Experimental details

Crystal data	
Chemical formula	C_12_H_12_N_2_^2+^·2C_8_H_7_O_4_^−^
*M*_r_	518.51
Crystal system, space group	Monoclinic, *P*2_1_/*c*
Temperature (K)	90
*a*, *b*, *c* (Å)	8.543 (2), 20.729 (5), 7.7061 (17)
β (°)	114.898 (4)
*V* (Å^3^)	1237.8 (5)
*Z*	2
Radiation type	Mo *K*α
μ (mm^−1^)	0.10
Crystal size (mm)	0.58 × 0.25 × 0.02

Data collection	
Diffractometer	Bruker APEXII CCD
Absorption correction	Multi-scan (*SADABS*; Bruker, 2016 ▸)
*T*_min_, *T*_max_	0.552, 0.746
No. of measured, independent and observed [*I* > 2σ(*I*)] reflections	20958, 3650, 2905
*R*_int_	0.066
(sin θ/λ)_max_ (Å^−1^)	0.708

Refinement	
*R*[*F* ^2^ > 2σ(*F* ^2^)], *wR*(*F* ^2^), *S*	0.047, 0.132, 1.03
No. of reflections	3650
No. of parameters	181
H-atom treatment	H atoms treated by a mixture of independent and constrained refinement
Δρ_max_, Δρ_min_ (e Å^−3^)	0.53, −0.22

Computer programs: *APEX2* and *SAINT* (Bruker, 2016 ▸), *olex2.solve* (Bourhis *et al.*, 2015 ▸), *SHELXL* (Sheldrick, 2008 ▸), and *OLEX2* (Dolomanov *et al.*, 2009 ▸).

## full crystallographic data

### Crystal data

**Table:**

C_12_H_12_N_2_^2+^·2C_8_H_7_O_4_^−^	*F*(000) = 544
*M**_r_* = 518.51	*D*_x_ = 1.391 Mg m^−^^3^
Monoclinic, *P*2_1_/*c*	Mo *K*α radiation, λ = 0.71073 Å
*a* = 8.543 (2) Å	Cell parameters from 4917 reflections
*b* = 20.729 (5) Å	θ = 2.6–30.2°
*c* = 7.7061 (17) Å	µ = 0.10 mm^−^^1^
β = 114.898 (4)°	*T* = 90 K
*V* = 1237.8 (5) Å^3^	Plate, clear colourless
*Z* = 2	0.58 × 0.25 × 0.02 mm

### Data collection

**Table:**

Bruker APEXII CCD diffractometer	2905 reflections with *I* > 2σ(*I*)
φ and ω scans	*R*_int_ = 0.066
Absorption correction: multi-scan (SADABS; Bruker, 2016)	θ_max_ = 30.2°, θ_min_ = 2.0°
*T*_min_ = 0.552, *T*_max_ = 0.746	*h* = −11→12
20958 measured reflections	*k* = −29→29
3650 independent reflections	*l* = −10→10

### Refinement

**Table:**

Refinement on *F*^2^	Primary atom site location: iterative
Least-squares matrix: full	Hydrogen site location: mixed
*R*[*F*^2^ > 2σ(*F*^2^)] = 0.047	H atoms treated by a mixture of independent and constrained refinement
*wR*(*F*^2^) = 0.132	*w* = 1/[σ^2^(*F*_o_^2^) + (0.0606*P*)^2^ + 0.4983*P*] where *P* = (*F*_o_^2^ + 2*F*_c_^2^)/3
*S* = 1.03	(Δ/σ)_max_ < 0.001
3650 reflections	Δρ_max_ = 0.53 e Å^−^^3^
181 parameters	Δρ_min_ = −0.22 e Å^−^^3^
0 restraints	

### Special details

**Table:**

Geometry. All esds (except the esd in the dihedral angle between two l.s. planes) are estimated using the full covariance matrix. The cell esds are taken into account individually in the estimation of esds in distances, angles and torsion angles; correlations between esds in cell parameters are only used when they are defined by crystal symmetry. An approximate (isotropic) treatment of cell esds is used for estimating esds involving l.s. planes.
Refinement. H atoms attached to heteroatoms were freely refined isotropically. H atoms connected to carbon atoms were placed geometrically (C—H = 0.95 Å) and refined as riding atoms with *U*_iso_(H) = 1.2*U*_eq_(C).

### Fractional atomic coordinates and isotropic or equivalent isotropic displacement parameters (Å^2^)

**Table:**

	*x*	*y*	*z*	*U*_iso_*/*U*_eq_	
O2	0.24089 (12)	0.42238 (4)	0.25115 (13)	0.0198 (2)	
O3	0.56630 (12)	0.41986 (5)	0.42339 (13)	0.0225 (2)	
O1	−0.04982 (12)	0.36590 (4)	0.01841 (14)	0.0217 (2)	
O4	0.71834 (12)	0.35219 (5)	0.32767 (15)	0.0254 (2)	
N1	−0.02080 (14)	0.41143 (5)	−0.43034 (15)	0.0171 (2)	
C6	0.41360 (15)	0.34659 (6)	0.17054 (16)	0.0147 (2)	
C7	0.25353 (15)	0.37113 (5)	0.14897 (16)	0.0145 (2)	
C2	0.09975 (15)	0.34088 (6)	0.02102 (17)	0.0158 (2)	
C11	0.26852 (16)	0.47304 (6)	−0.16647 (17)	0.0173 (2)	
C8	0.57618 (16)	0.37522 (6)	0.31710 (17)	0.0175 (2)	
C5	0.41996 (16)	0.29391 (6)	0.06001 (18)	0.0185 (2)	
H5	0.528263	0.277301	0.073926	0.022*	
C13	−0.03901 (16)	0.45885 (6)	−0.32230 (18)	0.0179 (2)	
H13	−0.151365	0.470811	−0.337355	0.022*	
C9	0.13651 (17)	0.39427 (6)	−0.41449 (18)	0.0194 (2)	
H9	0.146696	0.360930	−0.493684	0.023*	
C3	0.10839 (16)	0.28915 (6)	−0.08833 (18)	0.0190 (2)	
H3	0.005280	0.269293	−0.176397	0.023*	
C14	0.41779 (16)	0.50517 (6)	−0.01775 (18)	0.0198 (2)	
H14	0.395078	0.536024	0.059892	0.024*	
C12	0.10275 (16)	0.49066 (6)	−0.18939 (18)	0.0188 (2)	
H12	0.087893	0.524298	−0.113955	0.023*	
C10	0.28331 (16)	0.42435 (6)	−0.28499 (18)	0.0194 (2)	
H10	0.393602	0.412167	−0.276192	0.023*	
C4	0.26890 (17)	0.26605 (6)	−0.06931 (18)	0.0207 (3)	
H4	0.273946	0.230930	−0.145981	0.025*	
C1	−0.20613 (18)	0.33220 (7)	−0.0933 (2)	0.0270 (3)	
H1A	−0.233819	0.337474	−0.229464	0.041*	
H1B	−0.300275	0.349769	−0.066656	0.041*	
H1C	−0.191431	0.286266	−0.060228	0.041*	
H2	0.357 (3)	0.4327 (11)	0.340 (3)	0.055 (6)*	
H1	−0.131 (3)	0.3851 (10)	−0.532 (3)	0.056 (6)*	

### Atomic displacement parameters (Å^2^)

**Table:**

	*U*^11^	*U*^22^	*U*^33^	*U*^12^	*U*^13^	*U*^23^
O2	0.0201 (5)	0.0218 (4)	0.0187 (4)	−0.0005 (3)	0.0094 (4)	−0.0056 (3)
O3	0.0197 (5)	0.0270 (5)	0.0186 (4)	−0.0036 (4)	0.0060 (4)	−0.0076 (4)
O1	0.0138 (4)	0.0248 (5)	0.0271 (5)	−0.0010 (3)	0.0092 (4)	−0.0032 (4)
O4	0.0142 (4)	0.0300 (5)	0.0291 (5)	−0.0030 (4)	0.0063 (4)	−0.0096 (4)
N1	0.0155 (5)	0.0192 (5)	0.0148 (5)	−0.0025 (4)	0.0047 (4)	0.0013 (4)
C6	0.0138 (5)	0.0171 (5)	0.0130 (5)	−0.0019 (4)	0.0055 (4)	0.0002 (4)
C7	0.0163 (5)	0.0159 (5)	0.0123 (5)	−0.0012 (4)	0.0068 (4)	0.0005 (4)
C2	0.0144 (5)	0.0185 (5)	0.0150 (5)	0.0002 (4)	0.0067 (4)	0.0022 (4)
C11	0.0168 (6)	0.0190 (5)	0.0144 (5)	−0.0017 (4)	0.0049 (4)	0.0029 (4)
C8	0.0160 (6)	0.0205 (6)	0.0150 (5)	−0.0035 (4)	0.0056 (4)	0.0002 (4)
C5	0.0162 (6)	0.0202 (6)	0.0195 (6)	0.0001 (4)	0.0080 (5)	−0.0016 (4)
C13	0.0163 (6)	0.0192 (5)	0.0177 (6)	0.0010 (4)	0.0066 (5)	0.0026 (4)
C9	0.0193 (6)	0.0223 (6)	0.0176 (6)	−0.0007 (5)	0.0088 (5)	−0.0008 (4)
C3	0.0163 (6)	0.0214 (6)	0.0170 (6)	−0.0031 (4)	0.0046 (5)	−0.0021 (4)
C14	0.0189 (6)	0.0209 (6)	0.0190 (6)	−0.0017 (4)	0.0074 (5)	−0.0016 (5)
C12	0.0183 (6)	0.0186 (5)	0.0186 (6)	−0.0010 (4)	0.0070 (5)	−0.0002 (4)
C10	0.0153 (6)	0.0242 (6)	0.0194 (6)	−0.0002 (4)	0.0079 (5)	0.0010 (5)
C4	0.0213 (6)	0.0204 (6)	0.0207 (6)	−0.0021 (5)	0.0092 (5)	−0.0063 (5)
C1	0.0159 (6)	0.0304 (7)	0.0335 (7)	−0.0028 (5)	0.0090 (6)	−0.0009 (6)

### Geometric parameters (Å, º)

**Table:**

O2—C7	1.3535 (14)	C5—H5	0.9500
O2—H2	0.96 (2)	C5—C4	1.3818 (18)
O3—C8	1.2618 (15)	C13—H13	0.9500
O1—C2	1.3713 (15)	C13—C12	1.3806 (17)
O1—C1	1.4297 (16)	C9—H9	0.9500
O4—C8	1.2753 (16)	C9—C10	1.3797 (18)
N1—C13	1.3389 (16)	C3—H3	0.9500
N1—C9	1.3451 (17)	C3—C4	1.4005 (18)
N1—H1	1.09 (2)	C14—C14^i^	1.330 (3)
C6—C7	1.4018 (16)	C14—H14	0.9500
C6—C8	1.4954 (16)	C12—H12	0.9500
C6—C5	1.3999 (16)	C10—H10	0.9500
C7—C2	1.4144 (17)	C4—H4	0.9500
C2—C3	1.3849 (17)	C1—H1A	0.9800
C11—C14	1.4672 (17)	C1—H1B	0.9800
C11—C12	1.3998 (18)	C1—H1C	0.9800
C11—C10	1.4020 (17)		
			
C7—O2—H2	105.9 (13)	C12—C13—H13	119.4
C2—O1—C1	116.90 (10)	N1—C9—H9	119.5
C13—N1—C9	120.72 (11)	N1—C9—C10	121.02 (11)
C13—N1—H1	121.6 (12)	C10—C9—H9	119.5
C9—N1—H1	117.7 (12)	C2—C3—H3	119.9
C7—C6—C8	119.67 (10)	C2—C3—C4	120.11 (11)
C5—C6—C7	119.79 (11)	C4—C3—H3	119.9
C5—C6—C8	120.50 (11)	C11—C14—H14	117.2
O2—C7—C6	121.91 (10)	C14^i^—C14—C11	125.64 (15)
O2—C7—C2	118.38 (10)	C14^i^—C14—H14	117.2
C6—C7—C2	119.68 (11)	C11—C12—H12	120.2
O1—C2—C7	115.39 (10)	C13—C12—C11	119.60 (12)
O1—C2—C3	124.89 (11)	C13—C12—H12	120.2
C3—C2—C7	119.71 (11)	C11—C10—H10	120.2
C12—C11—C14	118.72 (11)	C9—C10—C11	119.52 (11)
C12—C11—C10	118.00 (11)	C9—C10—H10	120.2
C10—C11—C14	123.27 (11)	C5—C4—C3	120.57 (12)
O3—C8—O4	123.73 (11)	C5—C4—H4	119.7
O3—C8—C6	119.12 (11)	C3—C4—H4	119.7
O4—C8—C6	117.15 (11)	O1—C1—H1A	109.5
C6—C5—H5	120.0	O1—C1—H1B	109.5
C4—C5—C6	120.07 (11)	O1—C1—H1C	109.5
C4—C5—H5	120.0	H1A—C1—H1B	109.5
N1—C13—H13	119.4	H1A—C1—H1C	109.5
N1—C13—C12	121.11 (11)	H1B—C1—H1C	109.5
			
O2—C7—C2—O1	−2.15 (15)	C8—C6—C5—C4	177.25 (11)
O2—C7—C2—C3	178.70 (11)	C5—C6—C7—O2	−179.29 (11)
O1—C2—C3—C4	−177.73 (11)	C5—C6—C7—C2	2.46 (17)
N1—C13—C12—C11	−0.18 (18)	C5—C6—C8—O3	−175.32 (11)
N1—C9—C10—C11	0.76 (18)	C5—C6—C8—O4	4.15 (17)
C6—C7—C2—O1	176.16 (10)	C13—N1—C9—C10	0.90 (18)
C6—C7—C2—C3	−2.99 (17)	C9—N1—C13—C12	−1.20 (18)
C6—C5—C4—C3	−1.40 (19)	C14—C11—C12—C13	−176.93 (11)
C7—C6—C8—O3	2.21 (17)	C14—C11—C10—C9	176.58 (11)
C7—C6—C8—O4	−178.32 (11)	C12—C11—C14—C14^i^	−178.18 (16)
C7—C6—C5—C4	−0.28 (18)	C12—C11—C10—C9	−2.06 (18)
C7—C2—C3—C4	1.34 (18)	C10—C11—C14—C14^i^	3.2 (2)
C2—C3—C4—C5	0.9 (2)	C10—C11—C12—C13	1.78 (18)
C8—C6—C7—O2	3.16 (17)	C1—O1—C2—C7	−173.35 (11)
C8—C6—C7—C2	−175.09 (10)	C1—O1—C2—C3	5.75 (18)

Symmetry code: (i) −*x*+1, −*y*+1, −*z*.

### Hydrogen-bond geometry (Å, º)

**Table:**

*D*—H···*A*	*D*—H	H···*A*	*D*···*A*	*D*—H···*A*
N1—H1···O4^ii^	1.09 (2)	1.45 (2)	2.5402 (15)	178 (2)
O2—H2···O3	0.96 (2)	1.64 (2)	2.5270 (14)	150 (2)
C13—H13···O2^iii^	0.95	2.52	3.1831 (16)	127
C13—H13···O3^ii^	0.95	2.57	3.2092 (17)	125
C12—H12···O1^iii^	0.95	2.46	3.3591 (17)	159

Symmetry codes: (ii) *x*−1, *y*, *z*−1; (iii) −*x*, −*y*+1, −*z*.
